# Making better maize plants for sustainable grain production in a changing climate

**DOI:** 10.3389/fpls.2015.00835

**Published:** 2015-10-06

**Authors:** Fangping Gong, Xiaolin Wu, Huiyong Zhang, Yanhui Chen, Wei Wang

**Affiliations:** State Key Laboratory of Wheat and Maize Crop Science, Collaborative Innovation Center of Henan Grain Crops, College of Life Science, Henan Agricultural University, Zhengzhou, China

**Keywords:** maize ideotype, drought and heat stress, changing climate, sustainable food production, maize production

## Abstract

Achieving grain supply security with limited arable land is a major challenge in the twenty-first century, owing to the changing climate and increasing global population. Maize plays an increasingly vital role in global grain production. As a C4 plant, maize has a high yield potential. Maize is predicted to become the number one cereal in the world by 2020. However, maize production has plateaued in many countries, and hybrid and production technologies have been fully exploited. Thus, there is an urgent need to shape maize traits and architectures for increased stress tolerance and higher yield in a changing climate. Recent achievements in genomics, proteomics, and metabolomics have provided an unprecedented opportunity to make better maize. In this paper, we discuss the current challenges and potential of maize production, particularly in China. We also highlight the need for enhancing maize tolerance to drought and heat waves, summarize the elite shoot and root traits and phenotypes, and propose an ideotype for sustainable maize production in a changing climate. This will facilitate targeted maize improvement through a conventional breeding program combined with molecular techniques.

## Challenges and Potential of Maize Production

Achieving grain supply security with limited arable land will present a major challenge in the twenty-first century, owing to the growing world population and changing climate. The global population is expected to reach nine billion by 2050 (Shelden and Roessner, 2013), representing an additional two billion people to feed. The population in China is expected to peak at 1.6 billion in 2030. The world’s grain supply is inadequate compared with the food demand. Furthermore, changes in climate, particularly drought and extreme temperatures, will be more frequent in the near future and will severely limit crop production worldwide (Cooper et al., 2014; Frey et al., 2015; Horton et al., 2015).

**FIGURE 1:**
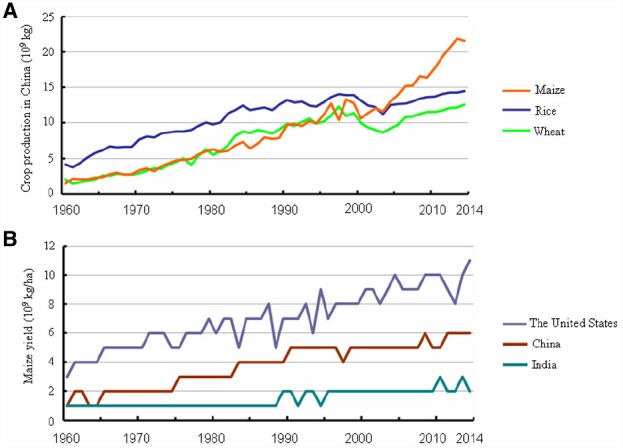
**Changes in maize yield from 1960 to 2014 in China, India, and the United States. (A)** Average annual production of maize, rice, and wheat in China. **(B)** Comparison of maize yield per unit area among China, India, and the United States. Data were retrieved at: http://www.indexmundi.com/.

Maize originated in the highlands of Mexico approximately 8,700 years ago (Piperno et al., 2009) and then spread to the Americas, Europe, and Asia primarily via trade networks. Maize is currently the second most plentiful crop globally (Ort and Long, 2014) and the largest crop produced in China, as a result of both larger planting area and greater yield (Figure 1A). Maize is predicted to surpass both wheat and rice to become the number one crop globally by 2020 (Jones, 2009). Theoretically, as a C4 plant, maize has a higher yield potential than wheat and rice. The maize grain yield per unit of land could be increased in developing countries. For example, maize yield per unit area in China is equivalent to about 2/3 of that in the United States, even lower in India (Figure 1B). As stated in the “National Grain Security and Mid- and Long-Term Planning Framework (2008–2020)” of China, a 50 billion kg increase in grain production is required by 2020 to ensure adequate grain supply, and maize will contribute 65% of this increased grain yield. Therefore, maize will play an increasingly vital role in grain production in China and worldwide.

Long-term domestication has radically changed maize from its origins. The morphology of modern maize is strikingly different from that of its wild ancient form (teosinte). Unlike teosinte, maize has hundreds of exposed kernels attached to a cob that is completely covered by husks and cannot reproduce on its own (Shi and Lai, 2015). In the last century, maize yields and quality in most maize-producing countries have been greatly increased through conventional hybrid breeding programs. Due to the common application of hybrid seeds and advanced management technologies, maize production has reached a plateau in many countries. Thus, for sustainable maize production, there is an urgent need to shape maize traits and architectures to cope with the challenges of increasing population and changing climate as well as the requirement of maize mechanization, as in other major crops (rice, Luo, 2010; wheat, Munns et al., 2010; legumes, Araújo et al., 2015). Maize improvement will benefit from a better understanding of genetic control of yield and stress endurance. Recent researches in *Arabidopsis* and crop plants integrated with genomics, proteomics, and metabolomics have accelerated the discovery of genes and molecular markers of important agronomic traits, which can potentially be used for genetic engineering and targeted breeding, respectively, of superior maize varieties. So, it is an unprecedented opportunity to make a better maize ideotype for higher yields and increased stress endurance in a changing climate.

In this paper, we discuss the current challenges of maize production, particularly in China. We also highlight the need for improvement of maize tolerance to drought and heat waves and propose traits and phenotype requirements for a maize ideotype. This information will assist in targeted maize improvement through molecular breeding approaches in the near future.

## Enhancing Maize Tolerance to Drought and Heat Waves

Drought and heat are two major environmental stressors and often occur concurrently. They severely limit plant productivity worldwide. In the near future, global climate change is likely to increase the risk of extreme temperature, particularly heat waves (Horton et al., 2015). Although maize originated in the tropics, it is highly sensitive to drought and heat, particularly at silk emergence and/or when flowers are ready for pollination (Boyer and Westgate, 2004; Lobell et al., 2011, 2014; Frey et al., 2015). In China, approximately 60% of the maize planting area is prone to drought and heat waves, which may result in as high as 30% yield loss in an affected year. Hence, enhancing maize tolerance to drought and heat is critical for sustainable maize production.

Creating drought- and heat-tolerant maize cultivars adapted to different geographical environments has proven to be a difficult task for maize breeders. As early as the 1970s, the International Maize and Wheat Improvement Center (CIMMYT) began a breeding approach for drought-tolerant maize lines and spent more than 30 years developing several drought-tolerant varieties (Ashraf, 2010). Drought- and heat-resistant maize varieties are still unavailable in other parts of the world. The responses to drought and/or heat stresses in plants are complex physiological and biochemical processes and involve changes in anatomic structures. These characteristics are determined by sets of genes within the maize genome. Thus, it is necessary to identify stress-related genes/proteins.

After stress-related genes/proteins have been identified, maize stress tolerance can be easily enhanced via genome editing technology. This process requires less time than a conventional breeding program. The availability of maize genomic sequencing and high-throughput genomics, proteomics, and metabolomics approaches have accelerated the identification of potential stress-related genes/proteins/metabolites in maize plants. A range of stress response-related proteins, such as heat shock proteins (HSPs), phytohormone regulators, signaling proteins, and protective enzymes, etc. (Huang et al., 2012; Gong et al., 2014; Yin et al., 2014; Hu et al., 2015a,b) contribute to drought and heat stress tolerance in maize. Protein sHSP26 improves chloroplast performance under heat stress by interacting with specific chloroplast proteins in maize (Hu et al., 2015b). The over-expression of trehalose-6-phosphate phosphatase in maize ears increased the concentration of trehalose and sucrose in ear spikelets, and improved eventual maize yield especially under severe drought condition (Nuccio et al., 2015). The predicted integral membrane proteins, encoded by *ARGOS* gene family, are novel negative regulators of ethylene signal transduction. In transgenic maize plants, over-expression of *ARGOS* reduced plant sensitivity to ethylene, leading to enhanced drought tolerance and greater grain yield under both drought stress and well-watered conditions (Shi et al., 2015).

Recently, Alter et al. (2015) developed the DroughtDB database (http://pgsb.helmholtz-muenchen.de/droughtdb/), which is an expert-curated compilation of plant drought stress genes and their homologs in nine model and crop plant species (including maize). Thus, DroughtDB facilitates the identification, analysis and characterization of genes involved in drought stress tolerance in agriculturally important crop plants. Major genes/proteins associated with drought and heat tolerance can be candidates for maize stress resilience improvement by transgenesis.

Maize root architecture must be shaped with the changing climate in mind. In maize, root cortical aerenchyma varies among genotypes and can be induced by hypoxia conditions (e.g., flooding) and suboptimal nutrient availability (e.g., N, P, and S deficiency; Bouranis et al., 2006; Mano et al., 2006). The formation of root cortical aerenchyma, which converts living cortical tissue to air volume, is beneficial in maize drought tolerance because it reduces the metabolic cost of soil exploration (Zhu et al., 2010). Furthermore, for maize roots, a reduced living cortical area (Jaramillo et al., 2013), reduced cortical cell file number (Chimungu et al., 2014a), and increased cortical cell size (Chimungu et al., 2014b) are potential traits that could improve maize drought tolerance. Additionally, maize genotypes with reduced lateral root branching density have superior water capture, growth, and yield under drought (Zhan et al., 2015). Thus, a phenotype with a few but long lateral roots is another selection target (Zhan and Lynch, 2015).

Maize leaf architecture may also need to be shaped with the changing climate in mind. In maize leaves, a larger number of stomata and shorter distance between the vascular bundles in the leaf blade are beneficial characteristics for drought tolerance (de Souza et al., 2013). Moreover, leaf curl, which helps conserve water for use in other functions, is an anatomical characteristic and can potentially be used to enhance plant tolerance to drought and heat. In maize, leaf curl is found to be controlled by few genes (Entringer et al., 2014). Thus, maize lines with rapid leaf-curling could be a useful resource for tolerance improvement. In the near future, shaping root/leaf architecture will be the key targets for improving maize in a changing climate.

## Shoot Traits and Phenotype Requirements for Sustainable Maize Production

Globally, new land for maize production is limited. Thus, it is essential to increase maize productivity per unit area to cope with the increased demand for grains. High-density planting is a practical approach for increasing maize yield per unit area. In China, the average maize yield is approximately 6,000 kg/ha with a planting density of 52,500–67,500 plants/ha, both of which are lower than those in the United States. If maize planting density in China increases by 15,000 plants/ha, the total maize yield is predicted to increase by 20%. However, maize varieties that are suitable for high-density planting are still lacking in some developing countries due to specific climates and environments; thus, breeding such varieties is an important task in maize improvement.

Multiple leaf-related traits of maize are related to high-density planting. Leaf angle is an important agronomic trait (Li et al., 2015). The preferred leaf angle of a maize plant follows a pattern of upper < middle < lower. A smaller leaf angle results in a more upright leaf orientation. This is beneficial for increasing the leaf area index, reducing maize shade syndrome and improving photosynthetic efficiency (Sakamoto et al., 2006). The quantitative trait loci for leaf angle were recently mapped (Ku et al., 2010, 2011, 2012; Li et al., 2015). Leaf angle-related genes, such as *ZmCLA4* (Zhang et al., 2014), were cloned and could be used in maize breeding for high-density planting and to maximize the maize yield.

The level of mechanized maize production is relatively lower in developing countries than in the United States. In China, the Yellow River Valley is the primary maize production area and often encounters rainy weather during the maize harvest season. Approximately 1/3 of the maize in this area is still picked by hand. Manual harvesting is time consuming and labor intensive. Furthermore, China has undergone rapid urbanization in recent years, and there were more people living in urban areas (690 million) than in rural areas (656 million) at the end of 2011. At that time, approximately 51% of the population lived in urban areas, compared to 26% in 1990. As a result, rural labor is extremely lacking. Therefore, the future development of maize production relies largely on the full implementation of agricultural mechanization in developing countries (e.g., China).

To meet the demands of mechanized harvest, several maize shoot traits should be shaped via both genetic improvement and molecular markers selection breeding. Maize kernels with a rapid filling speed, rapid dehydration and easy threshing characteristics are needed. Uniform and moderate plant and ear height, as well as lodging resistance upon maturity will also be beneficial characteristics for maize production mechanization. In China, a few promising maize varieties for mechanized harvest have been developed and are being evaluated in field conditions prior to large-scale planting.

## Root Traits and Phenotype Requirements for Sustainable Maize Production

Roots provide the interface between plants and the complex soil environment. Roots extract water and minerals contained in soils that are required for plant growth. Therefore, root architecture is particularly critical in water and nutrition acquisition. Over the past 100 years, maize breeding has focused on yield and shoot traits and phenotypes (York et al., 2015). Maize root architecture can now be modified for efficient water and nutrition use as well as to accommodate high-density planting.

Water uptake by roots is essential for plant growth and for high yields, particularly in water-deficit conditions. In an ideal maize root system, the primary roots run straight into the soil, the brace roots and crown roots are steep, and the angles of the brace roots are shallower than those of the crown roots. Such root traits can enhance water uptake from a drying soil, reduce root lodging and allow high-density planting and better growth of maize. Moreover, anatomical and molecular traits contribute to root performance. For example, reduced xylem vessel diameter can save soil water for later use by reducing the root hydraulic conductance (Meister et al., 2014); water channels in the roots can be modulated by controlling the expression of related-proteins/genes, such as aquaporin (Hachez et al., 2012). The water uptake of the above-mentioned root architecture traits must be intensively evaluated in drought-prone regions. Additionally, QTL mapping analysis has been used to reveal regions of the maize genome that control root architecture in water uptake (Landi et al., 2010; Cai et al., 2012; Zurek et al., 2015), and the cloning of relevant genes will facilitate the elucidation of the molecular mechanisms of root architecture and provide candidate genes for maize improvement.

Increasing nutrient uptake efficiency from soils is a major challenge for roots because of the wide range of soils in which maize is cultivated. The universally limiting nutrients in agricultural soils include N, P, and K. In many developing countries, a common practice in maize production is to apply excess chemical fertilization for high yield. This inevitably results in environmental degradation. According to the World Bank, China accounts for approximately 30% of global fertilizer consumption. Therefore, there is a need for the breeding and cultivation of maize varieties with increased tolerance to N/P/K deficiency.

An ideotype called “steep, cheap and deep” has been proposed for the maize root system, which represents steep and deep roots, and reduction of the metabolic cost of soil exploration. This system integrates architectural, anatomical, and physiological phenes (Lynch, 2013). N and water are highly mobile resources and enter the deeper soil strata. A root system with a rapid exploitation of deep soil would optimize N capture and water uptake in drying and N-deficient soils (Lynch, 2013). Other ideal root traits for N acquisition under N deficiency conditions include reduced maize lateral root branching (Zhan and Lynch, 2015) and smaller crown root number (Saengwilai et al., 2014). P is immobile and concentrates in the topsoil. Thus, the elite maize root traits and phenotype associated with enhanced P acquisition include shallow axial root growth angles, short but many laterals, and long root hairs (Lynch, 2013). To achieve an N/P acquisition balance in maize, an optimal lateral root branching density depends on N and P availability in soils (Postma et al., 2014). Additionally, cortical aerenchyma in roots can enhance maize growth and development in soil with N/P/K deficiencies (Postma and Lynch, 2010, 2011). Studies on maize root adaptation to N/P/K deficiency have recently been extended using proteomic, transcriptomic, and metabolomic approaches (Li et al., 2007; Simons et al., 2014; Abdel-Ghani et al., 2015; Trevisan et al., 2015). These approaches could provide substantial genetic resources for the improvement of maize root architecture. At present, the genes underlying root architecture and root adaptation to nutrition deficiency have not been characterized and cloned, and the molecular mechanism remains unclear.

In spite of the availability of multiple advanced breeding tools and identified elite trait-related genes, it is still difficult for maize breeding, because current genetic modification technology lacks the power to resolve drought tolerance as a single, broadly applicable solution. In many cases, maize yield will not synergistically increase with stress resistance. Thus, substantial work is needed before the genes can be used in maize breeding, particularly, the phenotypes of transgenic maize needs to be evaluated under filed and stressed conditions.

## Maize Ideotype for Targeted Maize Improvement

The term “ideotype” is defined as “a combination of morphological and/or physiological traits, or their genetic bases, optimizing crop performance to a particular biophysical environment, crop management, and end-use” (Martre et al., 2015). Making a maize ideotype with increased stress resistance, adaptation to high-density planting and improved efficiency of water and N/P/K acquisition from soil will be the most important concerns for maize improvement and sustainable grain production.

**FIGURE 2:**
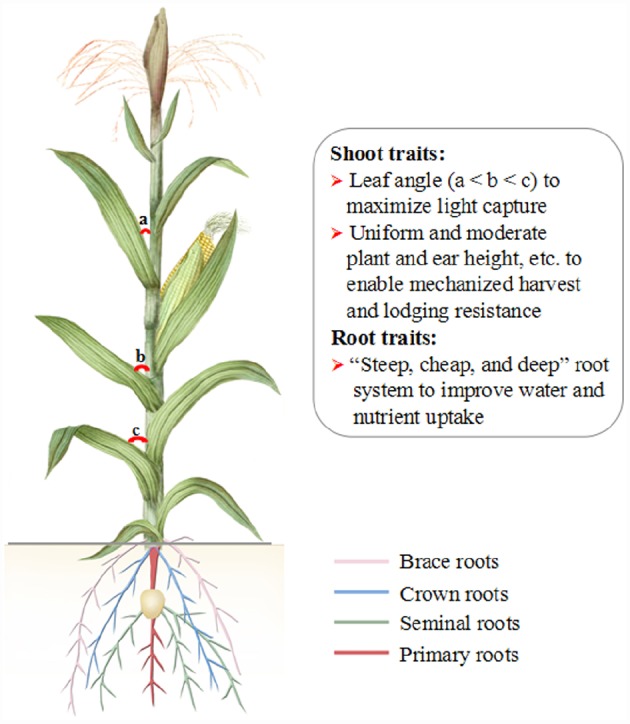
**A diagram of proposed maize ideotype.** Maize ideotype plants will have improved shoot and roots traits and phenotype, enhanced stress resistance and maintain a high productivity in a changing climate.

An ideotype of maize plants can be proposed based on recently relevant studies and the above discussion (Figure 2). A maize ideotype includes elite shoot traits (e.g., small leaf angle, fast kernel filling and kernel dehydration) and root traits (e.g., a “steep, cheap, and deep” root system). Moreover, an ideotype root has reduced living cortical area, reduced cortical cell file number, and increased cortical cell size in response to drought stress. Importantly, stress-related genes will be brought in transgenic maize plants for (biotic and abiotic) stress resistance and synergic high yield. The maize ideotype with above traits would allow to plant more plants per unit area, facilitate mechanized harvest, and improve water and nutrient uptake (especially in drying and nutrient-deficient soils).

## Conclusion and Perspective

Globally, maize production is entering a pivotal period when modern biotechnology is sufficiently powerful to make better maize plants for sustainable grain production in a changing climate. The maize ideotype proposed here may shed light on targeted maize improvement in the near future.

Increasingly promising genetic resources for elite maize traits are being discovered using high-throughput omics approaches, particularly genomics, proteomics and metabolomics. The application of these genetic resources in breeding practices can significantly increase the gene pools and allow for the modification of many important agronomic traits in maize. Conventional breeding programs combined with molecular modification techniques (e.g., gene transfer and genome editing) will greatly accelerate the speed of the creation of maize ideotypes. The creation of maize ideotypes and their subsequent application in production may play a vital role in ensuring sustainable grain production in a changing climate.

### Conflict of Interest Statement

The authors declare that the research was conducted in the absence of any commercial or financial relationships that could be construed as a potential conflict of interest.
